# American Medical Association

**Published:** 1869-03-01

**Authors:** Jas. F. Hibberd

**Affiliations:** Richmond, Ind.


### *American Medical Association.*

RICHMOND, IND., Feb. 19, 1869.

EDITOR CHICAGO MEDICAL JOURNAL:

In Washington, in May last, after the American Medical Association had decided to hold its next session in New Orleans, I promised several gentlemen to ascertain whether an arrangement could be made with a first-class steamer to take all those who might wish to go via the Mississippi from Cairo to New Orleans, on special terms. My correspondence warrants me in saying that such an arrangement can be made, and that the particulars can be published on the 1st of April. Meanwhile, I thought it might be of interest to your readers to know that such an arrangement was on the tapis.

The time from Cairo to New Orleans will not exceed four days, and the fare will not exceed $35 from Cairo to New Orleans and return. This includes meals and state-rooms. Possibly better terms can be made, but as soon as the best are obtained I will advise you.

Prof. Gross and others thought that if all who desired to go to New Orleans via the river, could be accommodated on one well-appointed boat, it would make the voyage pleasant, and perhaps profitable. My idea is that some general remark upon the subject in the editorial pages of your March issue might interest your readers.

Very truly, etc.,

JAS. F. HIBBERD.

